# Dietary Supplement Use among Non-athlete Students at a Canadian University: A Pilot-Survey

**DOI:** 10.3390/nu12082284

**Published:** 2020-07-30

**Authors:** Dalia El Khoury, Joel Hansen, Madelyn Tabakos, Lawrence L. Spriet, Paula Brauer

**Affiliations:** 1 Department of Family Relations and Applied Nutrition, University of Guelph, 50 Stone Road, Guelph, ON N1G 2W1, Canada; jhanse01@uoguelph.ca (J.H.); mtabakos@uoguelph.ca (M.T.); pbrauer@uoguelph.ca (P.B.); 2 Department of Human Health and Nutritional Sciences, University of Guelph, 50 Stone Road, Guelph, ON N1G 2W1, Canada; lspriet@uoguelph.ca

**Keywords:** nutritional supplements, prevalence, determinants, health behavior

## Abstract

Despite the emerging evidence of adverse consequences and interaction with doping substances, dietary supplements (DS) are commonly used by many Canadians. The purpose of this study was to evaluate the patterns and determinants of current DS use among non-athlete students at a Canadian university using a cross-sectional approach. Of the 475 participants who completed the online survey, 43.4% declared using DS in the past six months. Participants who were male, aged ≥20 years old, and had a parent/guardian with a bachelor’s degree were significantly more likely to use DS. The types of DS used and the sources of information regarding DS were significantly influenced by age and gender. The most commonly used DS were vitamin and mineral and protein supplements. Most participants referred to healthcare professionals for information on DS, but many continued to depend on unreliable sources including family and friends. Of DS users, 10.1% reported experiencing adverse events from using DS. Findings from this study indicate that supplementation is very common among Canadian non-athlete students and highlight the urgent need for the development of educational programs surrounding DS use.

## 1. Introduction

The wide availability of dietary supplements (DS) in the marketplace, combined with a renewed interest in personal health, has contributed to a steady annual increase in DS sales and use, with global sales reaching $128 billion in 2017 [1,2,3]. According to Binns et al. [3], many are under the impression that DS have benefits equal to or even greater than healthy diet and lifestyle. This is a risky mindset to have when the safety and efficacy of DS rely heavily on the strength of the scientific evidence and the quality control measures employed by manufacturers [3]. The term DS is generally defined as an orally consumed product that aims to supplement one’s diet [4]. Many products are considered DS, including vitamins, minerals, herbs, fish oils, probiotics, amino acids, and fiber supplements [4,5,6,7]. To date, most of the DS research has been conducted on athletes and general populations [7,8]; often overlooking the explicit examination of subpopulations such as college and university students despite their reported high prevalence of DS use in the United States [9,10]. In the Canadian general population, 45.6% of individuals were reported to regularly use at least one DS [7], and 40.1% of Canadian adults specifically take a vitamin and mineral supplement [11]. However, certain populations, such as university/college students in the United States, have exhibited higher supplementation rates (up to 80%) in studies to date [9]. It has been recognized that rates of supplementation in college populations vary with gender, age, physical activity patterns, body mass index (BMI), and tobacco usage [9,10,12,13,14]. Indeed, studies exploring physical activity and DS have shown that DS use among adults is generally more prevalent in active individuals [9,10,11,12,14,15].

One may assume the high prevalence of DS is beneficial as it may indicate a more “health-conscious” mindset. However, it was reported that these supplements were often inappropriate choices to address possible nutrient deficiencies and can increase nutrient intakes to levels associated with harmful outcomes [16]. Also, DS are known to advertise impressive health claims that are generally not supported by robust evidence [17].

Regardless of the potential health risks, people continue to depend on DS information from unreliable sources. It has been repeatedly shown that friends and family are the most preferred source of information regarding DS, and many fail to acknowledge the essential role of healthcare professionals in guiding the use of DS [9,14,16,18,19]. Additionally, as per the Gateway Theory, a developmental hypothesis formulated to model how adolescents initiate and progress in the use of various drugs [20], this increasing familiarity with supplement use could be worrying. There is evidence that the use of nutritional supplements, especially by individuals who strongly believe in their effectiveness, predicts the use of more dangerous and often controlled or illegal substances such as anabolic steroids [21,22]. Furthermore, up to 12–58% of commercially available supplements were found to contain potentially dangerous products not listed on the label, including hormones, prohormones, beta2-agonists, stimulants, and anabolic steroids [23,24,25,26].

In addition to risks of contamination, incorrect product dosing, supplement–supplement interactions, supplement–drug interactions, allergic reactions [6], cardiovascular complications, gastrointestinal distress, anxiety, fever, and nausea are commonly reported adverse effects of DS use in the general population and undergraduate students [12,27]. Additionally, Geller et al. [27] found that the US receives approximately 23,000 emergency department visits annually due DS, and 28% of patients are young adults aged 20 to 34 years old. Not surprisingly, 12–15% of Canadians have also reported adverse effects from taking supplements [6,28], although this number is likely a gross underestimate as only 41% of Canadians who experienced unwanted side effects to natural health products have reported them [6].

Despite the possible risks, and lack of clear government guidelines, many Canadians continue to use DS [7] to promote overall health, prevent illness, supplement the diet, lose adipose tissue, enhance muscle recovery, increase energy, and improve physical performance [2,9,12,14,15,16]. The motivations to consume DS and the popular sources of DS-related information have been well documented in the general North American population [2,7,28,29,30,31,32]. However, DS intake frequency, duration, types, and volume, along with the reasons for use and the common sources of information, are not well studied in typical Canadian (non-athlete) university students [12,32].

Analyzing self-reported data collected from university students at a university in Ontario, Canada, this research explored the supplementation patterns in this population and if supplement use habits were associated with their physical activity level. The objectives of this study were to identify (1) the volume, frequency, and duration of DS use, (2) the popular sources of DS information, (3) the reasons for DS use, and (4) the effects of lifestyle and demographic traits as predictors of DS use among non-athlete university students.

## 2. Materials and Methods

### 2.1. Participants and Recruitment

During the study period, 24,191 students were enrolled at the University of Guelph, the majority were undergraduate students (88.0%) and identified as female (59.0%) [33]. This study included 484 participants who self-identified as “non-athletes” and were full-time or part-time, undergraduate or graduate students, at the University of Guelph. Students who were “varsity athletes” were excluded from this study. Nine participants did not identify their supplementation status and were excluded from all analyses. Subsequently, the data from 475 questionnaires were analyzed, which resulted in a 98.1% response rate. Individuals with missing data were only excluded from the specific relating analyses, and no gender analysis was performed specifically on those with undisclosed gender to ensure participant anonymity. Participants were recruited based on a convenience sampling method via announcements made by the researchers and professors in three lectures, each containing 275, 400, and 600 students. The recruitment document which contained the survey URL was also posted eight times on the University of Guelph’s online learning management system by professors, where it was available to a total of 3127 students. Accordingly, the sample size of the pool of students from which participants were recruited was 4402. The questionnaire (Figure S1) required an adequate literacy of English and was completed by participants after signing an electronic informed consent form. After completing the questionnaire, participants had the option of submitting their email addresses, in a separate form, in exchange for a $5 gift card. The gift card website was not linked to the anonymous data collected through the survey.

This study was approved by the University of Guelph Research Ethics Board (REB#18-08-007), and data collection took place throughout September and October 2018.

### 2.2. Questionnaire Development and Design

The supplement use questionnaire was tested for content validity and reliability in the target population [34]. It consisted of multiple-choice response options, Likert scale-based responses, and open-ended responses. The questionnaire was composed of three sections: (1) physical activity patterns, (2) DS usage, and (3) demographic factors. At the beginning of the questionnaire, participants were asked “Are you a varsity athlete?” Those who self-identified as non-varsity athletes were the focus of the study and proceeded to the first portion of the questionnaire, which assessed physical activity levels using six questions from the International Physical Activity Questionnaire (IPAQ) [35]. Participants recorded the frequency and duration that they participated in vigorous physical activity, moderate physical activity, and walking in the past week. According to the IPAQ scoring protocol, metabolic equivalent (MET) values of 8.0, 4.0, and 3.3 were assigned to vigorous physical activity, moderate physical activity, and walking, respectively [36]. The assigned MET values were multiplied by the participant’s minutes per week of each activity to calculate MET-minutes per week. Participants were classified as “active” if they accumulated ≥600 MET-minutes per week and “sedentary” if they accumulated <600 MET-minutes per week [35]. According to the World Health Organization (WHO), “adults aged 18–64 should do at least 150 min of moderate-intensity aerobic physical activity throughout the week or do at least 75 min of vigorous-intensity aerobic physical activity throughout the week or an equivalent combination of moderate- and vigorous-physical activity” to maintain health [37]. When this recommendation is multiplied by the IPAQ scoring protocol, it results in a cut-off value of 600 MET-minutes per week. In the DS portion of the questionnaire, participants were asked “Have you consumed a dietary supplement in the past 6 months?” Dietary supplement users were asked to identify the types of DS they used in the past 6 months from a list of supplements based on an in-depth scan of the Ontario supplement market as well as the current literature. The list included 15 supplement categories with 55 individual supplements, and participants were given the opportunity to add any supplements that were not listed. The list excluded prohibited substances banned by the World Anti-Doping Agency (WADA), such as anabolic steroids and other illegal hormones and drugs, but these were briefly examined later in the survey. The volume (number and types of DS used in the past six months), frequency (consumption of DS in a typical week), and duration (overall length of DS use) were analyzed through a series of questions related to each category and subtype of supplement used. In addition, adverse reactions to DS, sources of DS-related information, reasons for DS use, reading DS nutrition labels, and participants’ learning interest with respect to DS were also examined. The last section of the questionnaire requested demographic information on the participant’s gender, age, ethnicity, parents/guardians’ education, smoking status, alcohol use, and self-reported weight and height. For clarification purposes, definitions were included in the questionnaire. The section of the questionnaire related to DS can be found in supplemental materials.

### 2.3. Statistical Analysis

Statistical analyses were conducted using Statistical Package for the Social Sciences (SPSS) version 26.0, and statistical significance was determined at *p* ≤ 0.05. Descriptive and frequency statistics were used to describe the sample characteristics based on gender, age, activity status, ethnicity, and other demographic aspects. Chi-square (χ^2^) tests of independence and Fisher exact tests were used on the data from supplement users to examine associations between gender, age, activity status, ethnicity, parents/guardians’ education, alcohol use, smoking status, and supplementation patterns. The subcategories of any significant demographic polychotomous variables were regressed together to determine which of these sub-categories were significantly associated with DS use. Data normality was tested by using the Shapiro–Wilk and Kolmogorov–Smirnov tests. Univariate and multivariate logistic regression was performed to assess associations between participants’ demographic and health-related traits and DS use. The possibility of interactions between variables was also explored.

## 3. Results

### 3.1. Participant Characteristics

Of the 475 participants who completed the survey, the majority were female (88.1%), Caucasian (77.5%), and physically active (96.3%) (Table 1). Males accounted for 11.2% of the sample, and 0.6% of individuals preferred not to disclose their gender or identified as other. Very few participants were classified as sedentary (3.7%). The participants’ age ranged from 18 to 31 years old, and the mean age was 20.1 ± 1.8 years old. The most frequently reported level of parents’/guardians’ education was a bachelor’s degree (34.3%) (Table 1).

### 3.2. Prevalence of Supplementation

Overall, 43.4% of participants reported using DS in the past 6 months. Based on Chi-square and logistic regression of individual factors, being ≥20 years old (*p* = 0.001), parents’/guardians’ education (*p* = 0.002), and being male (*p* = 0.05) significantly predicted DS use (Table 2). No first-order interactions were significant. When all potential predictors were regressed into one model, age (*p* = 0.001), gender (*p* = 0.019), and parents’/guardian’s education remained significant (*p* = 0.006) with no interactions. A secondary series of logistic regression analyses were performed to determine the specific level of parents’/guardians’ education that predicted DS use. The results indicated that a parent/guardian having a bachelor’s degree (β = 0.738; *p* = 0.005) significantly predicted DS use.

### 3.3. Types of Dietary Supplements

**Figure 1:**
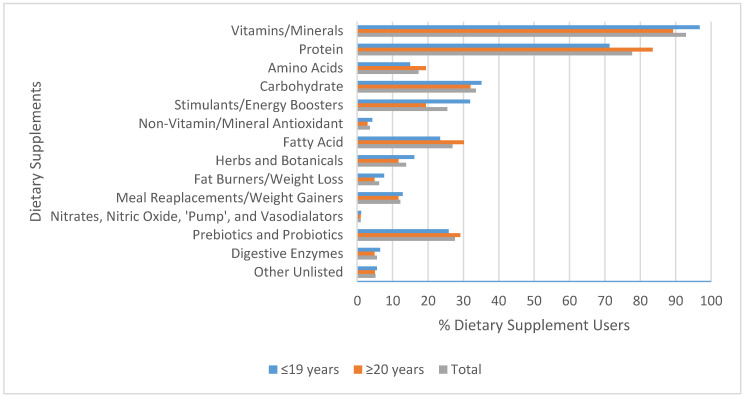
Percentage (%) of supplement users using different dietary supplements based on age (*n* = 94 for ≤19 years; *n* = 103 for ≥20 years; and *n* = 197 for total).

Overall, the most commonly consumed DS were vitamin/mineral supplements (93.5%) and protein supplements (77.6%). Nitrates, nitric oxide, “pump”, and vasodilators (1.0%) and non-vitamin/mineral antioxidants (3.5%) were the least commonly consumed DS. Age, gender, smoking status, and alcohol use all had significant associations with the specific types of DS used. Dietary supplement users aged ≤19 years old were more likely to use vitamin and mineral supplements (96.8% vs. 89.3%) (*p* = 0.041) and stimulant/energy booster supplements (31.9% vs. 19.4%) (*p* = 0.044) compared with those aged ≥20 years old. However, participants aged ≥20 years old were more likely to consume protein supplements (83.5%) compared with younger participants (71.3%) (*p* = 0.040) (Figure 1). Male DS users were significantly more likely to use amino acid supplements (36.7% vs. 13.6%) (*p* = 0.002) and stimulants (40% vs. 23.1%) (*p* = 0.05) compared with females. However, females were significantly more likely to use vitamin and mineral supplements compared with males (94.7% vs. 83.3%) (*p* = 0.041). Current smokers were more likely than previous smokers and nonsmokers to use amino acid (50.0% vs. 37.5% vs. 14.7%) (*p* = 0.021) and weight loss supplements (33.3% vs. 12.5% vs. 4.4%) (*p* = 0.020).

When participants were separated by activity status (sedentary vs. active), there were no significant differences in the types of DS consumed. The majority of active DS users reported using vitamins/minerals (93.3%) and protein (77.3%) supplements. Similarly, sedentary DS users reported a high usage of vitamins/minerals (100%) and protein supplements (85.7%).

### 3.4. Frequency and Duration of Use

Across all participants, the most common frequency of supplementation was ≤1 time per week (32.3%). The most common duration of DS use was ≥6 months (60.9%), which indicates that the majority of DS users engaged in long-term supplementation practices. There were no significant differences in the weekly supplementation rate and duration of DS use when comparing active and sedentary participants.

### 3.5. Reasons for Dietary Supplement Use Source of Information

**Figure 2:**
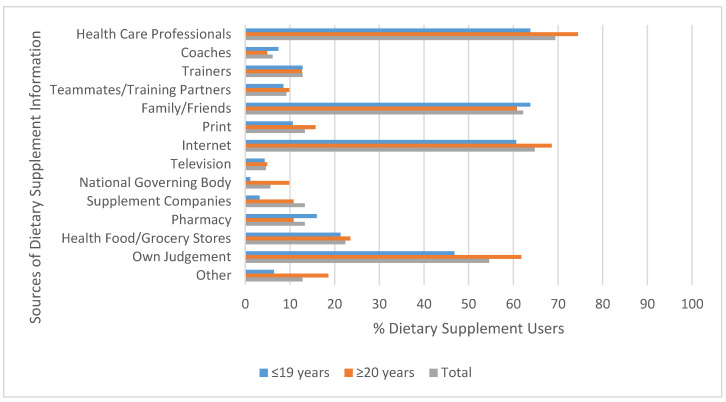
Percentage (%) of supplement users referring to sources of dietary supplement information based on age (*n* = 94 for ≤19 years; *n* = 102 for ≥20 years; *n* = 196 for total).

The most frequently reported reasons for using DS were to “maintain good health”, “increase energy”, “promote recovery”, and “lose weight”. There were no significant differences in reasons for DS use when participants were separated by age, gender, and activity status. Among DS users, the top sources of DS-related information were healthcare professionals (69.2%), the internet (65.2%), family and friends (61.7%), and their own judgement (53.7%). Age and gender had significant associations with the sources of information DS users referred to. Participants aged ≥20 years old were significantly more likely to use their own judgement (61.8% vs. 46.8%) (*p* = 0.05), refer to supplement companies (10.8% vs. 3.2%) (*p* = 0.039), and the national governing body (9.8% vs. 1.1%) (*p* = 0.008) for DS-related information (Figure 2). Females were significantly more likely to consult healthcare professionals about DS (73.4% vs. 51.1%) (*p* = 0.019). However, males were significantly more likely to refer to their trainers (31.0% vs. 8.9%) (*p* = 0.003) and teammates (24.1% vs. 5.9%) (*p* = 0.005) for information on DS. There was no significant interaction between activity status and sources of DS information as both groups referred to very similar sources. Among active and sedentary participants, healthcare professionals, family and friends, their own judgement, and the internet were the most frequently listed sources of DS information.

### 3.6. Nutrition Labels and Negative Side Effects

Overall, the majority of DS users (79.1%) expressed that they regularly read the nutrition labels on DS. Additionally, 15.9% reported to sometimes read nutrition labels, and 5.0% claimed to never read the nutrition labels. The most common reason for not reading nutrition labels included trusting the source of information and having sufficient knowledge about DS (58.5%). There were no significant differences in the tendency to read nutrition labels when participants were separated by their activity status.

Overall, 10.1% of DS users reported experiencing negative side effects from DS. The most common negative side effects were gastrointestinal issues, which were reported by 7.0% of DS users.

### 3.7. Non-Dietary Supplement Use

Only 0.9% of supplement users reported using non-DS performance-enhancing products such as steroids, prohormones, peptides, and ephedrine.

### 3.8. Participants’ Learning Interest

The majority of participants declared an interest in learning more about DS (91.7%). Of those interested, 25.7% were particularly interested in learning more about vitamin/mineral and protein supplements. Additionally, 14.7% of participants were interested in expanding their general knowledge of all DS. The preferred means of receiving DS-related information was through the internet (62.0%), presentations (28.0%), and consultations with healthcare professionals (20.3%).

## 4. Discussion

The DS user rate in our sample was similar to the supplementation rate of 45.6% among the Canadian general population [7]. However, the prevalence of DS use in our sample was considerably lower than that in most of the available literature on university students, which reported a supplementation rate between 53.6% and 80.0% in the United States [9,10,12]. The lower prevalence of DS use may be due to underreporting supplementation, the presence of educational programs tailored to this population, and different DS habits between Canadian and American university students.

Age, gender, and parents’/guardians’ education were significant predictors of DS use in the current study. Specifically, males, participants aged ≥20 years old, and those with a parent/guardian with a bachelor’s degree had significantly higher supplementation rates. The finding that male-identifying participants are significantly more likely to use DS mirrors the results of some studies on post-secondary students [12,29] but not others who reported higher rates of DS use among females [13]. It also contrasts with the results from recent studies on the general North American population, which found that females are more likely to use DS [7,11,18,38]. However, this finding should be interpreted with caution as this study had a very low male participation rate. It could be hypothesized that our small sample of males were more heavily invested in DS use, which could account for the higher user rate. Consistent with findings on the general Canadian population [7] and university students [10,12,13,14,15], supplementation was found to increase with age. This finding aligns with the general trend of a more health-conscious mindset as individuals age. The findings from the regression analyses also support the current literature, suggesting that being in a household of higher education is associated with higher DS consumption due to the greater accessibility and a stronger desire for “healthfulness” [11,38,39]. Contrary to previous reports, this study did not find physical activity status to be a significant predictor of overall DS use [9,10,11,12,14,15], which may have been related to the high percentage of respondents who were achieving physical activity recommendations in this sample.

Similar to the Canadian general population, the most commonly consumed type of DS in this study was vitamin/mineral supplements [7,18,28]. Alongside vitamins and minerals, this sample also indicated high levels of protein supplementation. Similar results have been reported in studies by Valentine et al. [12], Lieberman et al. [10], and Jackson et al. [29], showcasing that both vitamin/mineral and protein supplements are very popular among North American post-secondary students. Some of the common reasons for using DS explicitly correlated with the most frequently used supplements. Vitamin and mineral supplementation can be attributed to “maintain good health” and protein supplementation could be related to “promote recovery”. The high prevalence of protein supplementation is very interesting considering that most North Americans obtain adequate amounts of protein from their diet alone [40]. One could attribute the high use of protein supplements to the popular trend of consuming high-protein products especially after workout sessions [41] and the growing sector of protein supplements in the DS market [42].

In the present study, it was also found that age younger than 19 years predicted vitamin/mineral use, which is unusual considering that the majority of young adults do not experience micronutrient deficiencies [16]. This finding may indicate a gap in the participants’ nutrition and DS knowledge. Also, participants aged ≤19 years old reported significantly higher intakes of stimulants. A similar finding was reported by Lieberman et al. [10] in a sample of college students. They hypothesized that the students were using high levels of stimulants to increase their mental activity and strengthen their academic performance due to the highly competitive academic environment of most institutions [10]. However, individuals should be cautious when taking stimulants, as a study by Geller et al. [27] found that energy-boosting products are one of the most common reasons for young adults to visit the emergency department due to DS-related side effects. A finding unique to this study is that participants aged ≥20 years old were significantly more likely to use protein supplements. As mentioned before, the majority of North American young adults consume sufficient amounts of protein [40], so the high prevalence of protein supplementation could reflect the general misinformed belief that protein intake above requirements will always have a beneficial impact on strength and performance. It was also found that male-identifying participants were significantly more likely than females to use amino acid and stimulant supplements. This finding was also reported by El Khoury et al. [43] among male exercisers in gyms in Beirut city, Lebanon. The high usage of amino acid supplements among males is not surprising since it has been reported that males tend to gravitate towards DS like amino acids that support muscle maintenance [10]. The finding that females were significantly more likely to use vitamin and mineral supplements than males has been previously reported [43]; this pattern was mainly attributed to adopting a “healthy behavior” and/or improving an inadequacy in dietary habits.

There were no significant differences in the weekly frequency of supplementation in relation to activity status. This may imply that regardless of physical activity patterns, DS users are following the manufacturer’s recommended dosing protocols.

The top reasons for using DS were “maintain good health”, “increase energy”, “promote recovery”, and “lose weight”, which matches previous findings on post-secondary students and the general North American population [2,9,12,14,15,16].

The prevalence of adverse effects associated with DS use was slightly lower than the 12–15% rate reported among the general Canadian population [6,28]. The most commonly reported negative side effect, gastrointestinal (GI) distress, has been previously described in a sample of post-secondary students [8]. In general, GI symptoms such as nausea, vomiting, and abdominal pain are fairly common side effects of overall DS use [27]. However, in this sample many participants reported experiencing GI symptoms after the use of protein supplements, specifically whey-based protein supplements. It has been reported that gastrointestinal distress is a common side effect of whey protein intake, especially in individuals with an allergy or sensitivity to dairy [44].

An intriguing result from this study was that healthcare professionals were one of the most frequently reported source of DS information. This finding disagrees with the findings of Perkin et al. [14] and Snyder et al. [31], who observed that very few individuals consulted health professionals for DS-related information. Nonetheless, in certain university samples, healthcare professionals have been observed as a popular source of DS information [10,12]. However, it was also found that many individuals referred to unreliable sources such as the internet, family, and friends, as well as their own judgement, which supports much of the current literature [9,10,12,14,16,18,19,29]. This trend is of great concern because the information spread via the internet and word of mouth is largely unregulated [29]. To ensure the health and safety of consumers, it is important that DS users seek information from reliable sources, including healthcare professionals (i.e., physicians and registered dietitians). As reported in the literature, this study found significant differences in the sources of DS-related information participants relied on based on their demographic characteristics. Participants aged ≥20 years old were significantly more likely to use their own judgement as a reason for DS use. This finding suggests a high perceived knowledge about DS but does not affirm the accuracy of this knowledge. As reported by Barry [18], many individuals self-prescribe DS because they think DS are “natural” and cannot have adverse effects. Contrary to this perception, there is evidence in the literature depicting alarming consequences of naïve DS use [6,27]. However, a positive trend for those aged ≥20 years old is that they were significantly more likely to refer to sources of DS information that were published by the government (i.e., Health Canada and Statistics Canada). Similar to the results of this study, findings by Lieberman et al. [10] indicated that males were significantly more likely to refer to their trainers and teammates for DS-related information. It has also been previously reported that females are more likely to consult healthcare professionals for information on DS [10,43].

To our knowledge, this study is the first of its kind in a Canadian university population. This research has filled a large gap in the literature, which ignored an important population of non-athlete university students who are also heavily using DS. Furthermore, other strengths of the current study include the extensive array of information collected and the use of a validated questionnaire that was tested on the target population. Findings from this study provide crucial knowledge for the development of educational programs that are focused on the safe and adequate use of DS and that target younger health-conscious adults who are at great risk for misuse of DS and advancement to the use of doping substances.

The limitations are inherent to the nature of the study. The data collected were self-reported and subject to risks such as social desirability and recall bias. Due to the nature of the data, it was impossible to guarantee honesty and accuracy. To overcome this, the consent form highlighted that participation was completely anonymous. Also, the convenience sampling method resulted in an uneven distribution of participant’s demographic and lifestyle characteristics. This created a nondiverse sample and thereby limited the generalizability of the results. Specifically, the low male participation rate greatly hinders the generalizability of the findings with respect to gender differences in DS use. Further research should examine representative samples by creating a sampling frame that accurately reflects the population’s gender, activity status, age, and ethnicity distribution. This would ensure that both the common and exceptional experiences of individuals are reported and reduce the presence of bias. A representative sample could be better achieved through means of quota sampling, or other recruitment methods. Although the study assessed DS habits over the past six months, it was still cross-sectional in nature and only provided a snapshot of the true supplementation practices of participants at one time point. Future research should use longitudinal designs to better explore the patterns of DS use over the life course. Further research should also be done on the dietary habits of individuals in relation to DS use to support or negate previous reports that DS choices made by Canadians are often inappropriate. It has been expressed that DS users naturally eat healthier diets that fulfill their needs for macronutrients and micronutrients even before taking DS [16,45]. However, Payette and Gray-Donald [46] found that among DS users, the nutrients lacking in their diet were also lacking in the supplementation. Another limitation to this study was the volume of incomplete submissions. Although the missing data were excluded from the corresponding analyses, the end-results would have been more accurate if the whole dataset was complete. Finally, this study was conducted at a single Canadian university in southwestern Ontario. As a result, the findings may not be generalizable to the rest of the country.

## 5. Conclusions

This research helps fill large gaps in the literature surrounding DS use in the Canadian university setting. It provides the first look into DS use by non-athlete Canadian university students and raises the bar for the quality and rigor of research in this area of study. The results of this study help provide a more complete understanding of DS use in young adults who are non-athletes and its association with demographic and lifestyle characteristics. Results from this study support the consensus that approximately one in two Canadians regularly use DS across the lifespan. Although many participants referred to healthcare professionals for DS-related information, a considerable number sought information from unreliable sources such as the internet and friends and family. Despite the lack of scientific rigor and potential of adverse effects, post-secondary students continue to exhibit a high DS-user rate. Given the ready availability and accessibility of DS, further research and regulation are urgently needed to ensure consumer’s safety, appropriate product selection, and manufacturing accuracy. This research acts as a starting point to guide future studies on Canadian DS consumption patterns and their determinants and would support the development and implementation of educational programs surrounding DS use.

## Figures and Tables

**Table 1:** Descriptive characteristics of participants.

Characteristics	Prevalence
**Supplement Use (*n* = 475)**	
Yes	43.4%
No	56.6%
**Participant Age (*n* = 456)**	
Mean ± SD:	20.1 ± 1.8
Range	18–31
**Age Groups (*n* = 456)**	
≤ 19	59.4%
≥ 20	40.6%
**Gender (*n* = 464)**	
Female	88.1%
Male	11.2%
Other/Prefer not to disclose	0.6%
**Ethnicity (*n* = 475)**	
Caucasian	77.5%
South East Asian	8.0%
South Asian	6.7%
West Asian	0.2%
Black	4.4%
Indigenous	2.3%
Latin	2.3%
Arab	2.1%
Prefer not to disclose	1.1%
Other	2.9%
**Parents’/Guardians’**	
**Education (*n* = 475)**	
Bachelors	34.3%
Diploma	28.8%
College	27.4%
High School	17.3%
No Diploma	2.7%
Other Education	1.9%
**Activity Status (*n* = 461)**	
Active	96.3%
Sedentary	3.7%

*n*: number of participants.

**Table 2:** Logistic regression analysis of independent predictors of dietary supplement use.

Variable	*n*	B	S.E.	Significance	Exp(B)	95% C.I. for EXP(B)	
						Lower	Upper
**Age**	**475**	**0.569**	**0.172**	**0.001**	**1.766**	**1.262**	**2.472**
**Parent/Guardians’ Education**	**461**	**0.162**	**0.053**	**0.002**	**1.176**	**1.060**	**1.305**
**Gender**	**461**	**0.583**	**0.297**	**0.050**	**1.791**	**1.001**	**3.203**
Alcohol	425	0.287	0.239	0.229	1.333	0.835	2.128
Smoking	435	0.458	0.613	0.456	1.580	0.475	5.259
Ethnicity	463	−0.003	0.036	0.934	0.997	0.929	1.070
Activity Status	461	0.103	0.502	0.837	1.109	0.414	2.965

*n*: number of participants; S.E.: standard error; C.I.: confidence interval; significant at *p* ≤ 0.05 (bolded).

## Supplementary Materials

The following are available online at https://www.mdpi.com/2072-6643/12/8/2284/s1, Figure S1: Dietary supplement-related section of the questionnaire.
