# Supplementary material for: Dietary Supplement Use among Non-athlete Students at a Canadian University: A Pilot-Survey
Source: Nutrients. 2020 Jul 30;12(8):2284. doi: 10.3390/nu12082284 (PMC7468929; doi:10.3390/nu12082284)
Supplement: Supplementary file 1 [file nutrients-12-02284-s001.pdf]

UNIVERSITY of GUELPH

DEPARTMENT OF FAMILY RELATIONS AND APPLIED NUTRITION
College of Social and Applied Human Sciences

APPENDIX F

Questionnaire

This survey aims to determine the prevalence and potential determinants of dietary supplement use among university students with different exercising habits. **This survey is anonymized** and will not be linked to your name or other unique identifiers. **The data that are collected through this questionnaire will be kept confidential**. Participation in completing this questionnaire is voluntary. You are free to decline to answer any questions you do not wish to answer, or stop participating at any time. It should take you approximately 15 minutes to complete this survey.

Section 2: Dietary Supplements

Dietary supplements are defined as "an orally consumed product intended to supplement one's diet".

1. Have you consumed a dietary supplement in the past 6 months?

- ☐ Yes **(If yes, please proceed to questions 2 to 7)**
- ☐ No **(If no, please proceed to question 8)**

For each of the categories of dietary supplements listed below (a-n), the following questions will be asked individually for each category:

a. **Vitamins/Minerals**
   - Vitamin D
   - Vitamin C
   - Vitamin B12
   - Iron
   - Calcium
   - Multi-vitamin/multi-mineral supplements
   - Other(s). Please specify ________

b. **Protein**
   - Whey protein
   - Casein protein
   - Soy protein
   - Creatine
   - Protein bars/powder/shakes
   - Other(s). Please specify_______

c. **Amino Acids**
   - Glutamine
   - Amino acids blend
   - BCAA (branched chain amino acids)
   - L-leucine
   - Other(s). Please specify_______

d. **Carbohydrate**
   - Sports drinks
   - Gels
   - Powders
   - Other(s). Please specify __________

e. **Stimulants/Energy Boosters**
   - Energy drinks
   - Pre-workout supplements
   - Caffeine pills
   - Others(s). Please specify__________

f. **Non-Vitamin/Mineral Antioxidants**
   - Food polyphenols (e.g., quercetin, açaí)
   - CoQ10
   - Glutathione
   - Other(s). Please specify_______

g. **Fatty Acids**
   - Omega-3
   - CLA (conjugated linoleic acid)
   - Fish oil
   - Other(s). Please specify ______

h. **Herbs and Botanicals**
   - Gingko biloba
   - Ginseng
   - Echinacea
   - Natural testosterone boosters
   - Other(s). Please specify __________

i. **Fat Burners/Weight Loss**
   - Diuretics
   - Garcinia cambogia
   - Green coffee bean extract
   - Green tea extract
   - L-carnitine
   - MCT oil/powder
   - Other(s). Please specify __________

j. **Meal Replacements/Weight Gainers**

k. **Nitrates, Nitric Oxide, 'Pump', and**

FINAL

Vasodilators (e.g., beetroot juice or powder, l-arginine, and citrulline malate)

l. **Prebiotics and Probiotics**
m. **Digestive enzymes**
n. **Other unlisted supplement(s) Please specify_____________________**

2. Have you consumed (insert category of dietary supplement (a-n)) supplements (insert examples within category) at any time in the past 6 months?

- ☐ Yes **(If yes, please proceed to questions 3 to 5 of that category)**
- ☐ No **(If no, please proceed to the next category)**

3. On average, how many times in a typical week do you consume (insert category of dietary supplement (a-n)) supplements?

- ☐ **Insert example(s) within category of dietary supplement and repeat for each example individually**
  - o Don't use
  - o ≤ 1 time per week
  - o 2-3 times per week
  - o 4-5 times per week
  - o ≥ 6 times per week

4. For how long have you been using (insert category of dietary supplement (a-n)) supplements?

- ☐ **Insert example(s) within category of dietary supplement and repeat for each example individually**
  - o < 1 month
  - o 1-2 months
  - o 3-5 months
  - o ≥6 months

5. Please specify your reason(s) for using (insert category of dietary supplement (a-n) supplements:

- ☐ **Insert example(s) within category of dietary supplement and repeat for each example individually**
  - o Correct or prevent micronutrients deficiencies
  - o Maintain good health
  - o Improve immunity
  - o Improve mood and/or decrease stress
  - o Correct or prevent diseases
  - o Supply convenient forms of energy and/or macronutrients
  - o Serve as meal replacement
  - o Enhance competitive performances
  - o Increase energy
  - o Increase alertness and mental activity
  - o Support intense training regimens
  - o Promote recovery
  - o Alleviation of musculoskeletal pain
  - o Lose weight/decrease fat mass
  - o Gain weight
  - o Increase or maintain muscle mass
  - o Financial gain (sponsorship)
  - o "Just in case" insurance policy
  - o Know or believe that others (e.g., friends, athletes, competitors) use this supplement
  - o Other(s). **(If checked off for any example, the question below will be asked)**

Please specify your other reason(s) for using (insert category of dietary supplement (a-n):

____

6. What is/are your source(s) of your information regarding dietary supplements in general (check all that apply)?

- ☐ Health care professionals (e.g., physicians, team physicians, specialists, dietitians, sports nutritionists)
- ☐ Coaches
- ☐ Trainers
- ☐ Teammates or training partners
- ☐ Friends/Family
- ☐ Print (e.g., magazines, books)
- ☐ Internet
- ☐ Television
- ☐ National governing body
- ☐ Supplement companies
- ☐ Pharmacies
- ☐ Health food/Grocery stores
- ☐ My own judgment
- ☐ Other(s). Please specify _________________

FINAL

Rank your sources of information regarding dietary supplements from the most used to the least used ones.

____

______________________________________.

7. Do you tend to read the labels (nutrition facts, ingredients, etc.) on your dietary supplements?

- ☐ Yes **(If yes, please proceed to question 8)**
- ☐ No **(If no, please proceed to If "No" or "Sometimes", why?)**
- ☐ Sometimes **(If sometimes, please proceed to If "No" or "Sometimes", why?)**

If "No" or "Sometimes", why?

- ☐ I trust my source of information/I already know enough about my supplements
- ☐ I do not know how to read food labels
- ☐ I do not care about reading food labels
- ☐ No specific reason

8. To your knowledge, have you ever used any of these non-dietary supplements, anabolic steroids, injectable peptides, amphetamines, pro-hormones (steroid analogues) or ephedrine, in an exercising context?

- ☐ Yes. Please specify ____________________ ____________________________________
- ☐ Not to my knowledge
- ☐ Prefer not to disclose

FINAL
